# ID 143 - Identificação de Boas Práticas na Decretação de Obsolescência de Dispositivos Médicos: ênfase em equipamentos

**DOI:** 10.5327/2237-9622.2025.v34s1.143

**Published:** 2025-11-25

**Authors:** Telma Vinhas Cardoso, Geovanna Martins de Moura, Daiana Lanrenci Orth Blas

**Keywords:** dispositivo médico, equipamento médico, ciclo de vida, obsolescência, boas práticas

## Abstract

**Introdução:**

A necessidade de retirada e substituição de equipamentos médicos (EM) do parque tecnológico de instituições de saúde tem sido apontada pelos setores técnicos pelo acompanhamento do ciclo de vida dos EM, geralmente com o uso de indicadores de desempenho. A ausência de metodologias que definam quais indicadores apontam a etapa de obsolescência de uma tecnologia dificulta a identificação desta última fase do seu ciclo de vida, de modo a não só gerar gastos excessivos com manutenção e substituição de componentes, mas também comprometer a qualidade do serviço prestado. A carência por documentos que auxiliem a reconhecer o período de obsolescência de um equipamento motivou o desenvolvimento deste trabalho, onde, por meio da elaboração de uma estratégia de levantamento de dados, pretende-se verificar a existência de sistemáticas que apontem a adoção de boas práticas no processo de decretação de obsolescência.

**Método:**

Identificou-se, na literatura e nos termos MESH via BVS, tanto os conceitos associados à obsolescência de equipamentos como um conjunto de palavras-chave que foram utilizadas para levantamento de documentos em bases de dados, tais como Google Acadêmico, PubMed, Biblioteca Virtual em Saúde e Embase nos últimos cinco anos. Foram definidos critérios de inclusão como ano de publicação (janela temporal abrangendo os últimos cinco anos), status da instituição de saúde (sendo privilegiados documentos de agências estatais e hospitais com reconhecimento internacional) e o idioma português.

**Resultados:**

Foram obtidos cinco resultados cujo conteúdo abordava meios de auxiliar a identificação de tecnologias obsoletas. Quatro deles foram identificados como sendo trabalhos acadêmicos desenvolvidos em Universidades Brasileiras, enquanto um deles foi desenvolvido em Portugal. Buscas não apontaram nenhuma política nacional relacionada à decretação da obsolescência em EM. Também não foi possível localizar nenhuma evidência de adoção dessa prática por unidades hospitalares públicas ou privadas. Por meio das buscas realizadas nas plataformas digitais de agências internacionais de ATS, foi possível identificar um restrito número de metodologias adotadas para a identificação e a decretação de obsolescência em tecnologias da saúde em quatro países: Espanha, Canadá, Malásia e Itália.

**Conclusão:**

Entre os quatro países onde foram identificadas metodologias para abordar essa última fase do ciclo de vida de uma tecnologia, apenas dois deles apresentaram uma documentação que define indicadores a serem adotados para elaboração de um relatório de obsolescência. Desses dois resultados, apenas um demonstrou intenções de infundir a metodologia no sistema de saúde. O pequeno número de processos de avaliação dos EM no final do seu ciclo de vida pode ser justificado pela pouca disponibilidade de diretrizes para identificação de tecnologias obsoletas. A etapa de obsolescência, no entanto, não dever ser vista como um processo menos importante do que a incorporação, visto que também é uma medida de prevenção de gastos. Sendo assim, a reavaliação de uma tecnologia em utilização deve ser vista como um processo a ser iniciado não apenas quando há evidências de que uma inovação concorrente entrará no mercado. Para reduzir a complexidade intrínseca ao problema, destaca-se a necessidade em se desenvolver políticas e práticas de identificação da obsolescência nos EM, a partir da elaboração de medidas fundamentadas na coleta de evidências obtidas das diferentes estratégias adotadas mundialmente.

